# Supplementary material for: Role of Diagnostics in Epidemiology, Management, Surveillance, and Control of Leptospirosis
Source: Pathogens. 2022 Mar 24;11(4):395. doi: 10.3390/pathogens11040395 (PMC9032781; doi:10.3390/pathogens11040395)
Supplement: Supplementary file 1 [file pathogens-11-00395-s001.zip › pathogens-1649654-supplementary.pdf]

Supplementary Information for: Role of diagnostics in epidemiology, management, surveillance, and control of leptospirosis

Authors: Sykes, J.E.; Reagan, K.L.; Nally, J.E.; Galloway, R.L.;
Haake, D.A.

Journal: *Pathogens* **2022**, *11*, 395. https://doi.org/10.3390/pathogens11040395

Supplemental Table S1. MAT Panel Serovars

Centers for Disease Control and Prevention	California Animal Health and Food Safety Laboratory at the University of California-Davis	National Veterinary Services Laboratories
Serum source: Humans<br><br>Serovars:<br><br>*L. interrogans* serogroup Australis serovar Australis<br>*L. interrogans* serogroup Australis serovar Bratislava<br>*L. interrogans* serogroup Autumnalis serovar Autumnalis<br>*L. borgpetersenii* serogroup Ballum serovar Ballum<br>*L. interrogans* serogroup Bataviae serovar Bataviae<br>*L. interrogans* serogroup Canicola serovar Canicola<br>*L. weilii* serogroup Celledoni serovar Celledoni<br>*L. kirschneri* serogropup Cynopteri serovar Cynopteri<br>*L. interrogans* serogroup Djasiman serovar Djasiman<br>*L. kirschneri* serogroup Grippotyphosa serovar Grippotyphosa<br>*L. santarosai* serogroup Hebdomadis serovar Borincana<br>*L. interrogans* serogroup Icterohaemorrhagiae serovar Icterohaemorrhagiae<br>*L. interrogans* serogroup Icterohaemorrhagiae serovar Mankarso<br>*L. borgpetersenii* serogroup Javanica serovar Javanica<br>*L. santarosai* serogroup Mini serovar Georgia LT 117<br>*L. interrogans* serogroup Pomona serovar Pomona<br>*L. interrogans* serogroup Pyrogenes serovar Pyrogenes<br>*L. santarosai* serogroup Pyrogenes serovar Alexi<br>*L. interrogans* serogroup Sejroe serovar Wolffi<br>*L. borgpetersenii* serogroup Tarassovi serovar Tarassovi	Serum source: Companion Animals<br><br>Serovars:<br><br>*L. interrogans* serogroup Icterohaemorrhagiae serovar Icterohaemorrhagiae<br><br>*L. interrogans* serogroup Canicola serovar Canicola<br><br>*L. interrogans* serogroup Australis serovar Bratislava<br><br>*L. interrogans* serogroup Pomona serovar Pomona<br><br>*L. kirschneri* Serogroup Grippotyphosa serovar Grippotyphosa<br><br>*L. interrogans* Serogroup Sejroe serovar Hardjo	Serum source: Agricultural Animals<br><br>Serovars:<br><br>Limited panel:<br>*L. interrogans* sg Australis sv Bratislava<br>*L. interrogans* sg Canicola sv Canicola<br>*L. interrogans* sg Grippotyphosa sv Grippotyphosa<br>*L. interrogans* sg Icterohaemorrhagiae sv Copenhageni<br>*L. interrogans* sg Pomona sv Pomona<br>*L. interrogans* sg *Sejröe sv* Hardjo<br><br>Extended panel also includes:<br>*L. interrogans* sg Australis sv Australis<br>*L. interrogans* sg Autumnalis sv Autumnalis<br>*L. borgpetersenii* sg Ballum sv Ballum<br>*L. interrogans* sg Bataviae sv Bataviae<br>L. *kirschneri* sg Cynopteri sv Cynopteri<br>*L. interrogans* sg Djasiman sv Djasiman<br>*L. interrogans* sg Hebdomadis sv Hebdomadis<br>*L. interrogans* sg Mini sv Szwajizak<br>*L. interrogans* sg Pyrogenes sv Pyrogenes<br>*L. borgpetersenii* sg Sejröe sv Sejröe<br>*L. borgpetersenii* sg Tarassovi sv Tarassovi<br>*L. tipperaryensis* sg Not Designated sv Room 22

Supplemental Table S2: Modified Faine's criteria (2012)*

	Score
Part A: Clinical data	
Headache	2
Fever	2
Temperature > 39°C	2
Conjunctival suffusion	4
Meningismus	4
Myalgia	4
Conjunctival suffusion + Miningismus + Myalgia	10
Jaundice	1
Albuminuria/Nitrogen retention	2
Hemoptysis/Dyspnea	2
Part B: Epidemiologic factors	
Rainfall	5
Contact with contaminated environment	4
Animal contact	1
Part C: Bacteriologic and laboratory findings	
Positive Culture and/or PCR	25
Serology	
One or more of the following:	15
IgM-ELISA	
Slide agglutination test	
Other serologic test	
MAT – single positive titer ≥400	
MAT – paired sera with four-fold rise in titer	25
Diagnosis	
Confirmed: Positive Culture and/or PCR	
Presumptive	
Total of Parts A and B	≥26
Total of Parts A, B, and C	≥25
Possible	20-24

*Adapted from reference [1].

Supplemental Table S3: Human Leptospirosis Case Definitions*

Suspect Case: Acute febrile illness (≥38.5°C) and/or severe headache with:
- Myalgia
- Prostration and/or conjunctival suffusion, and
- History of exposure

Probable Case (clinical definition): Suspect case w/any **two** of the following:
- Calf tenderness
- Cough with or without hemoptysis
- Jaundice
- Hemorrhagic manifestations
- Meningeal irritation
- Anuria/oliguria and/or proteinuria
- Shortness of breath
- Cardiac arrhythmias
- Skin rash
- Abortion

Probable Case (laboratory definition)**:** Suspect case with + rapid IgM test and/or any 3 of the following:
- Urinary findings: proteinuria, white blood cells, red blood cells
- Relative neutrophilia (>80%) with lymphopenia
- Platelets <100,000/mm^3
- Elevated serum bilirubin (>2mg%); liver enzymes moderately raised

Confirmed Case: Suspect or probable case with any one of the following:
- Isolation of leptospires from clinical specimens
- Positive PCR result
- Seroconversion from negative to
  positive or 4-fold rise in MAT titer
- MAT titer ≥400 in a single sample

*Adapted from reference [2].

References

1. Kumar, S.S. Indian guidelines for the diagnosis and management of human leptospirosis. In *India: Medicine Update*; Muruganathan, A., Ed.; The Association of Physicians of India: Mumbai, India; 2013; pp. 23–29.
2. WHO. *Informal Expert Consultation on Surveillance, Diagnosis and Risk Reduction of Leptospirosis. Chennai, India, 17–18 September 2009*; SEA-CD-217; World Health Organization – Regional Office for South-East Asia: New Delhi, India. Available online: https://tinyurl.com/4kdnnsz7 (accessed on 03/23/2022).
